# The Hospital. Nursing Section

**Published:** 1907-03-30

**Authors:** 


### The Hospital.

**Nursing Section.**

Contributions for "THE HOSPITAL," should be addressed to the EDITOR, "THE HOSPITAL" NURSING SECTION, 28 & 29 Southampton Street, Strand, London, W.C.

No. 1,072.—VOL. XLI. SATURDAY, MARCH 30, 1907.

### Notes on News from the Nursing World.

### THE SEVENTH ORDER.

THE Nurses' Registration Bill is down for second reading on Tuesday, April 9, and some persons may be under the impression that there will therefore be a debate in Parliament upon the measure that evening. But as it is the seventh order on the list, the first order being the Territorial and Reserve Forces Bill, which is hotly opposed, there is not the slightest prospect that its promoters will be able to bring it on. In fact there is practically not the ghost of a chance that the Bill will make any further progress this session. An appeal has been addressed to asylum-trained nurses to sign a petition in favour of registration in a separate register, "in view of impending legislation"; but there is no occasion for the latter to hurry up. Another year will obviously do just as well.

### EXONERATION OF THE LATE MATRON OF SWANSEA HOSPITAL.

IT will be remembered that a few weeks ago we commented on a long discussion at a meeting of the Swansea Hospital Board, in the course of which the chairman of the meeting, Mr. Hughes, confessed that the authorities had been guilty of "sweating," and said that he was "actually ashamed of himself." These and other remarks appear to have been regarded by the late matron as a reflection upon her administration and general management; and the chairman of the House Committee, Colonel Morgan, has therefore written to her husband to say that it was far from the intention of any of the members who spoke to convey such an impression. He affirms, indeed, that the desirability of increasing the number of nurses, in order to do away with outside help, and to secure for the regular nursing staff the valuable experience and training they were thus losing, was brought to the notice of the House Committee "long ago." This is a complete exoneration of the late matron—if exoneration were needed. We note, too, that Colonel Morgan regrets the use of the word "sweating" by Mr. Hughes as very unfortunate, because, he thinks, "it probably applies to the Swansea Hospital only to the same extent as it does to others"; and he likewise affirms that his statement that since August not one of the night nurses had had a single day's holiday, ought to have been that "the night nurses did not have their proper nights off, no relief being available." We do not think that this part of Colonel Morgan's letter in any way alters the fact that the additions to the staff were urgently needed some time, at any rate, before they were made. The present matron has, in short, got the increase which the late matron ought to have had "long ago."

### THE MONTHLY PAYMENT OF SALARIES.

Two questions of interest were raised at the last meeting of the Preston Guardians. It was proposed that the nursing staff of the Fulwood Workhouse should be given a week-end off duty once each quarter in their turns, and that their salaries should be paid monthly instead of quarterly as at present. With regard to the former, it was contended that the nurses at Fulwood are better off than those in several other Lancashire Unions, and that their fortnight's holiday per year, their day and half-day per month, their off duty for four or six hours two evenings per month, and two to four hours other evenings, is sufficient. We are afraid that if a week-end once a quarter was also granted, the ratepayers would complain, even though the arrangement did not involve any additional cost. As to the monthly payment of salaries, there is much to be said in favour of it. The point against it is that it gives a good deal of extra trouble in account-keeping. If the more frequent payment is greatly desired, we do not think that this objection should be regarded as insuperable. The majority of the Preston Guardians, however, declined to make either of the concessions asked for, or to allow them to be referred to the Nursing Committee.

### NURSING AND HEALTH VISITING.

THE proceedings at the annual meeting of the Kendal Home Nursing Association were exceptionally interesting. Dr. John Robertson, medical officer of health for Birmingham, who moved the adoption of the report, and Miss Margaret le Boileau, who seconded, specially dealt from the point of view of practical experience with the proposal of the committee to extend the work to health visiting. Dr. Robertson said that for 12 or 13 years he had come more or less in daily contact with the work of health visitors appointed by the Corporation of Birmingham, and he was greatly impressed with the value of their labours. He described the work as very hard, and declared that unless the health visitor had plenty of recreation there would soon be a breakdown in her own health. She should be an educated lady, have sympathy with her work, and training of it. Miss Boileau, who has herself been health visitor in Wakefield for nearly three years, spoke of the "amazing ignorance" which she had to contend with, especially in the treatment of infants, and maintained that the best work a health visitor could do was to prevent children from being injured by ignorant treatment. The experiment of adding a health visitor to the staff at Kendal was supported by a generous legacy to the Association, and no doubt seems to be entertained that the nucleus thus provided will be adequately supplemented by subscriptions.

### POOR-LAW NURSES AND OUTSIDE DUTIES.

The Alton Guardians have very wisely consulted the Local Government Board in respect of the action of district medical officers in the Union, who have asked the nurses at the workhouse to undertake duties connected with the removal of outside cases to the institution. The reply of Mr. Baldwin Fleming is that neither district medical officers nor relieving officers have authority to requisition the services of workhouse nurses outside the workhouse. In these circumstances it may be hoped that the medical officers will in future refrain from making calls upon the nurses, which are contrary to the regulations, and which might easily form a serious addition to their obligations.

### TREASURY PROSECUTION OF A MENTAL NURSE.

The Bristol magistrates had before them last week the case of a mental nurse, formerly engaged at Fishponds Asylum, and now married, who was charged by the Lunacy Commissioners with having received a lunatic to board and lodge with her in unlicensed premises. The charge was all the more serious because the patient killed himself by swallowing spirits of salts when he was in her care. There was no question of the *bona fides* of the defendant. During the time she was at Fishponds Asylum she obtained several medals, and she had intended to qualify as a trained nurse. Possibly her marriage interfered with her intention. Her defence was that she believed that she had a right to receive the patient without a reception order. We share the surprise expressed by the chairman that, in spite of her connection with a well-known mental institution, she had so little knowledge of the provisions of the Lunacy Act of 1890, bearing in mind the probability that the life of the patient might have been preserved if he had been put into proper custody. The penalty of a fine of forty shillings shows that the bench took a lenient view of the offence. It cannot be too widely known that partially or fully trained nurses run a risk of heavy penalties when they receive into their home a person of unsound mind without a reception order from a magistrate.

### GUY'S NURSES' PHOTOGRAPHIC SOCIETY.

How pleasantly and profitably nurses can employ their leisure was shown on March 22, when the Guy's Nurses' Photographic Society held a display of the work of its members in the Nursing Home. Prizes were given for different sections by the Countess of Bective, Lady Henry Bentinck, Mrs. Cosmo Bonsor, and Mrs. Trevelyan Martin, while the Society itself provided others, including a special prize, which was won by Miss M. Smith for a beautiful picture of the Henrietta Raphael Nurses' Home. The same lady won a first prize for a graceful study of "Birch and Bracken." Miss Fisher gained the second prize with a very artistic study of a mist effect, "On the River." The dispersion of Guy's nurses over the world was borne witness to by the variety of scenes depicted. Nurse Wilkinson contributed a picture of an old Buddhist railing at Ammad Napma, Ceylon, and Nurse Bingham some interesting "Scenes in a Pilgrimage City" in India. Nurse Phillips's picture of "A Matron's Sitting-room" had a charm of another kind.

### SCOTTISH NURSES WITHOUT RECREATIONS.

At the last meeting of Edinburgh Parish Council a letter from the nurses at Craiglockhart Poorhouse was read, in which they complained of their working hours and other conditions of employment. Special mention was made of the failure to provide any form of recreation, and it was stated that there is not even a newspaper in the institution. The letter was referred to the House Committee, who drew up the rules to which the nurses take exception. We certainly think that something in the way of light reading might be provided, and that the nurses might at least be permitted the opportunity of reading the news of the day. Recreation in reason tends to better work in the wards and an increase of cheerfulness and patience.

### CERTIFIED MIDWIVES' DEFENCE UNION.

A general meeting of the Certified Midwives' Defence Union will be held on Friday, April 5, at 10 Adelphi Terrace, Robert Street, John Street, Strand, at 6 p.m., Dr. Stanley Atkinson in the chair. It is hoped that all midwives interested in this question and intending to join the Union will attend, as it is desired to at once form a thoroughly representative society.

### NORTH LONDON NURSING ASSOCIATION.

The annual meeting of the North London Nursing Association was held in the Northern Polytechnic. The Secretary, in reading the report, alluded to the great regret of all connected with the association, at the resignation of the Lady Superintendent, Miss Meyer, and of Miss de Luttichan, who had done such signal service for the past 29 years. Miss Lund, who has already been working in the Home for seven years, had been appointed to take Miss Meyer's place. The balance sheet showed a credit balance of £130 10s. 10d. A sale of work at the Home, organised by the nurses, brought in £111 10s., and a ball given by the Hon. Nina Ray Shuttleworth and Miss Shuttleworth in the West End realised £268. Several lodges of friendly societies took collecting-boxes, and in one very poor district more than 200 people contributed a farthing per week. There are ten nurses on the staff of the association, and during the year the number of cases nursed was 1,729, while the visits amounted to upwards of 35,000.

### AN INCIDENT AT SCARBOROUGH.

It is seldom that a nurse who is attending church is called upon to render professional assistance to a fellow-worshipper, and less often still that her services are needed by one of the officiating clergy. At Scarborough, on Sunday morning, a priest had conducted the blessing of the palms at St. Peter's Roman Catholic Church, and had commenced Mass when he fainted. Another priest at once went to his help, and a nurse who was present volunteered aid, which was gratefully accepted. The patient was removed to the adjoining house, where he remained in a semi-unconscious state for upwards of an hour, and then recovered.

### A NETWORK OF NURSES FOR LEEDS.

In his speech at the annual meeting of the Leeds District Nursing Association the Vicar of Leeds, referring to the heroism of Captain Sperling in saving the last of the survivors on the *Berlin*, said that he did not wish to underrate the work done on a great occasion, "but he did want the people of Leeds to remember that they themselves were living quietly side by side with those who, without public applause, were yet carrying out an enterprise which was hardly second in the amount of self-sacrificing labours entailed." This is a high estimate of the efforts of the nurses employed by the Association, and we hope that it will do much to convert the deficit of £48, with which the year ended, into a surplus. The visits paid by the staff in the twelve months exceeded 81,000, and at the dispensary the nurses in charge attended to nearly 20,000 patients. One of the speakers affirmed that the aim of the organisation is to have a network of nurses over the city, but this cannot obviously be attained without a substantial augmentation of financial support.

### THE CATHOLIC NURSES' ASSOCIATION.

We are asked to state that, though additional meetings of the Catholic Nurses' Association are being held alternately at 109 St. George's Road, Southwark, and 15a Vicarage Gate, Kensington, the organisation has not moved from its original headquarters at Harrow-on-the-Hill. Meetings are still held regularly at Harrow on the first Friday of each month.

### A CHALLENGE FROM WORKING MEN.

An exceedingly gratifying feature in the report of the Sunderland District Nursing Association, which was submitted at the annual meeting, was commented upon by the Chairman. There was a time when the working men of the town were open to the reproach of neglecting to support the Association adequately. But as in the past twelve months they subscribed the handsome sum of £634 18s. 6d., that reproach has been wholly removed; and we agree with the Chairman that this sum constitutes a challenge to the people who are more wealthy to come forward and give regular help to the organisation. As a matter of fact, the amount contributed by the working men was a little more than half the total, and though the Society has still in hand a balance of £150, it started the year with one of £261. The appreciation of the Association by the medical men of Sunderland is attested by the circumstance that they called in the assistance of nurses in upwards of a thousand cases. But it is of the utmost importance that the well-to-do classes should vie with the working men in their efforts to render the financial position sound.

### PRINCESS CHRISTIAN'S MATERNITY HOME.

The first annual report in connection with the Maternity Home at Windsor, founded by Princess Christian in February last year, has been issued. The staff of the Home consists of a matron in charge, who is assisted by two fully-qualified and certificated midwives. During the ten months from March 1 to December 31, 27 patients were admitted to the institution, and 269 were attended in their own homes. The work has been inspected by the Local Supervising Authorities for Berkshire and for Buckinghamshire, by the Central Midwives Board, and by the Inspector of the Jubilee Institute for Nurses, all of whom expressed themselves entirely satisfied with what they saw.

### QUEEN'S NURSES IN LIVERPOOL.

At the annual meeting of the Liverpool Queen Victoria District Nursing Association Mr. John Henderson, J.P., in proposing a vote of thanks to the staff, spoke of the Staff Benevolent Fund, and said he would have pleasure in giving £500 in order that the pensions to nurses might be augmented, as he did not think the amount now provided was anything like adequate. It may be hoped that Mr. Henderson's generosity will be emulated by some of his fellow-citizens. Mr. Archibald Williamson, M.P., who seconded the adoption of the report, dwelt on the progress of the Association since it took over the district nursing work from the Royal Infirmary in January 1898. At that time there were twenty-five nurses, and now there are fifty-seven. In the past year the nurses attended nearly 9,000 cases, and paid visits little short of 250,000, the number of individual cases treated being larger than the number similarly treated in the wards of any of the hospitals.

### A THOUSAND SIXPENCES FOR A DISTRICT NURSE.

Last month an appeal was made by Mr. Alderman House, of Bishop Auckland, for a thousand sixpences in aid of the work of Miss Wood, a nurse in the district. In a few weeks the whole of the sum was subscribed—a gratifying evidence of the high esteem in which Miss Wood is held. It is twelve years since she commenced her labours as district nurse, and it is significant that, though other organisations in the town have had difficulties owing to various causes, her services and those of her assistant are so much appreciated, not only among the poorest of the poor, but also receive warm recognition at the hands of the clergy and others who are in a position to judge of their value.

### SHORT ITEMS.

A meeting of the Nurses' National Total Abstinence League will be held at the London Temperance Hospital, Hampstead Road, on Saturday, April 27, at 3.30 p.m., when an address will be given by the Rev. Canon Barker. Any nurse who would like to attend can have a card of invitation on application to the Secretary of the Women's Total Abstinence Union, 4 Ludgate Hill, E.C.—An entertainment given last week by Dr. Hamilton Irving, a brilliant amateur whose lightning caricatures and comic sketches are very clever, resulted in the handsome sum of £53 10s. 5d. being handed over to the Huddersfield and District Victoria Sick Poor Nurses' Association.—A meeting of the Nurses' Social Union was held at Minehead on March 20, when Mrs. Yale Fairset gave an address on "The Influence of the Nurse in the Home."

### The Nursing Outlook.

"From magnanimity, all fears above;
From nobler recompense, above applause,
Which owes to man's short outlook all its charm."

### THE MIDWIVES BOARD AND POOR-LAW INSTITUTIONS.

The deputation which waited on Lord Crewe, Lord President of the Council, on Friday last included representatives of poor-law guardians, of various associations and societies interested in the training and supply of midwives, and of members of the medical profession. The object of the deputation was to protest against the setting up of two standards for the training of midwives. One the higher standard of the Central Midwives Board. The other that under a less efficient and strict system formulated by the Privy Council in regard to poor-law institutions at the instance of the Local Government Board. It is evident from Lord Crewe's reply to the deputation that he is lamentably ignorant—as for that matter, strange as it may seem, appears to be the Local Government Board itself—of the real conditions and inefficiency now actually existent in very many poor-law institutions scattered throughout the country. In order to bring out the truth clearly we will give one example of this inefficiency. An infirmary with under forty beds has one charge nurse, who is assisted by three girls or wardmaids, one of the latter taking charge of the patients in the absence of the trained nurse. Such wardmaids are frequently absolutely ignorant of nursing, having had no training whatever, so that the whole work and care of the patients throughout each twenty-four hours must pracically devolve upon one nurse. Sometimes it happens that the nurse has only one wardmaid to help her with 38 patients, most of whom need skilled nursing from the severe nature of their ailments. The medical officer pays a daily visit, but otherwise the nurse has the whole responsibility, and has to meet every emergency which may arise, the doctor being unavailable for very many hours when he is on his rounds miles away. One such nurse, to our knowledge, has had eighteen hours' work per day for a fortnight at a time, in addition to night calls, and she has only been outside the infirmary gates three times in four months. Sometimes, when there are severe cases, this solitary nurse has only two hours in bed out of the twenty-four, because she feels she must do her duty to her patients. Such a conditions of affairs is declared to be not uncommon in poor-law infirmaries of the inferior type, although it constitutes the gravest reflection on the humanity of the whole nation, is perilous to the best interests of the patients, and destructive of the health of the nurses employed in such work.

Lord Crewe, however, inferentially argues that, this state of affairs cannot exist under the Local Government Board, seeing that this Board "is responsible for its every act to Parliament, and everybody who took exception to anything done by them had a simple and easy means of calling them to account." Knowing that the condition of affairs of which we have given a striking instance prevails in certain poor-law institutions all over the country, we should be glad to know what Lord Crewe has to say in justification of so misleading a statement, having regard to its important bearing upon the welfare and lives of many of the poorest and most miserable of our countrymen and countrywomen? The fact is, that the Privy Council, unless the Lord President can be made to realise the true inwardness of the present position, by setting up two standards in regard to the training of midwives, will permit the abominations now existing to continue, as we have shown, under the supervision and control of the Local Government Board. We are confident however that Lord Crewe would have made a much more sympathetic reply to Friday's deputation had he known that the case he was supporting under the conditions as they exist to-day might involve the recognition of such an infirmary as that we have referred to. Surely Lord Crewe's sympathy in regard to institutions of this kind, cannot rest with the Local Government Board, when he realises that such a course entails the waiving of the inspection of these ill-managed institutions under satisfactory supervision? How is it that the Local Government Board have permitted guardians to carry on poor-law infirmaries under the disgraceful conditions we have just shown to exist? The answer must be found in the fact that the actual medical supervision exercised by the Local Government Board over poor-law institutions is by means of only two medical inspectors, one for London and one for the whole of England and Wales.

Lord Crewe's reply and the general attitude he has taken up in this matter indicate a very real danger to the whole community, due to the confident belief most people have that when a Government department is responsible to Parliament, nothing that is seriously wrong can long continue under its control. Friday's deputation and its reception afford an excellent test of the practical value of this belief. How many members of the House of Commons or the House of Lords will put down questions as to the present condition of certain poor-law infirmaries in this country, and the dangers which are likely to arise to the mothers of England by the setting up of two standards of training, and the setting aside of the authority conferred upon the Central Midwives Board by Parliament, in order to save the face of a department of the Government? Any public-spirited member of Parliament, who will take the trouble to master the facts, may profitably keep Lord Crewe, Mr. John Burns and their departments, in a state of continuous activity for the rest of the session, and so win the gratitude of many thousands of people all over the country.

### The Central Midwives Board and the Local Government Board.

### DEPUTATION TO THE LORD PRESIDENT OF THE COUNCIL.

A representative deputation arranged by the Association for Promoting the Training and Supply of Midwives on Friday, March 22, waited on the Earl of Crewe, Lord President of the Council, to lay before him certain points in connection with the suggestion to exempt Poor-law midwives from the rules of the Central Midwives Board. The deputation was introduced by Sir Dyce Duckworth, M.D., and in addition to those who spoke as representing various bodies, there were present: Miss Alice Gregory, to represent the Midwives' Institute; Miss E. S. Haldane, to represent the Workhouse Nursing Association; Mr. Alfred Goodrich, Chairman of the Midwives Act Committee, L.C.C., to represent the London County Council; Dr. Annie McCall; Dr. J. Middleton Martin, M.O.H., Gloucestershire; Dr. C. E. Paget, M.O.H., Northants; Dr. George Reid, M.O.H., Staffordshire; Mr. Raffles Hughes, K.C.; Mr T. Almond Hind; Mrs. Wallace Bruce, Chairman of the Executive Committee of the Association for Promoting the Training and Supply of Midwives; Lady Balfour of Burleigh; Miss Lucy Robinson; Miss Lorent Grant; Mr. A. L. Leon, and other members of the Executive Committee of the A.P.T.S.M.

Sir Dyce Duckworth said: I have the honour to present to your Lordship a deputation from the Association for Promoting the training and Supply of Midwives, and also representatives from other institutions, to direct attention to the importance of maintaining at its present high standard a uniform and trustworthy training of midwives in this country. For myself, I may say that I represent the Queen's Jubilee Nursing Institute, on which I sit, by the Queen's appointment, as the representative of the Royal College of Physicians, and the Chairman of the Midwives' Committee of the Institute. We are moved to address you by reason of reports which have reached us to the effect that the Local Government Board has it in comtemplation to organise a system of training for midwives of its own; one which, so far as we can foresee, is not likely to prove adequate or satisfactory, and not in accord with the requirements of the Central Midwives Board. We are naturally apprehensive of great disturbance arising in this country from a dual standard of training which would press hardly on those who have received that demanded by the Board already empowered to carry out this work, and would not, in the case of a less satisfactory training, secure the confidence of other bodies and the public, who regard the Central Midwives Board as the only one authorised by the State to lay down the essential requirements in this matter. Speaking for the Royal College of Physicians, I may state with all due respect that the College would unhesitatingly defer to the opinions of a body of experts, such as the Central Midwives Board, rather than to those of the Local Government Board in this case, and we see no reason why any exemption should be introduced into the matter of midwives' training in this country. The Queen's Jubilee Nursing Institute, at all events, could not recognise any midwives who have had an inferior training to that required by the Central Midwives Board; and as the Institute has now a large experience of this subject and is installed all over the country, any material change introduced into the standard of training must thus have widespread and serious consequences for the public benefit. Those who will now address your Lordship will enter more fully into particulars, and adduce facts which will show the grounds of our apprehensions in this matter. We can only bespeak a fair hearing of our objections, and trust to your Lordship's open-mindedness to judge as to their soundness. I am authorised by Princess Christian, President of the Workhouse Nursing Association, to say that she entirely endorses and supports the views to be laid before you, and would regard it as impossible that there should be two standards of training.

Dr. C. J. Cullingworth said: We have the honour to speak on behalf of the Association for Promoting the Training and Supply of Midwives, an association of which the Queen is the Patron and the Archbishop of Canterbury the President, which represents a large body of lay as well as medical opinion on the subject at issue. The association is desirous of submitting considerations with regard to certain proposed exemptions from the rules of the Central Midwives Board. The practical effect of these exemptions would be that all midwives being trained or holding appointments under the Poor-law authorities would be to a certain extent removed from the jurisdiction of the Central Midwives Board. The Central Midwives Board, for instance, whilst still retaining in its own hands the duty of examining all candidates for admission to the Midwives Roll, would no longer have complete control over the details of their education and practical training, which the association regards as of greater importance than the examination itself. The association desires, in the first place, to call attention to the provisions of the Midwives Act. That Act places the entire responsibility for the training and certification of the midwives of this country, without exception, upon a specially constituted Board, largely consisting of experts—that is, upon the Central Midwives Board. The association is advised that, whatever rules and bye-laws it may make to the contrary, the Central Midwives Board would continue to be legally responsible for the education and training of the candidates admitted to its examinations and for the regulation of their future practice. But whatever the law on the subject may be, the association is strongly of opinion that the introduction of two standards of training—one which would be under the full control of the Central Midwives Board and one which would not—would seriously hamper the Board in its useful work and greatly detract from the value of its certificate. The Board's certificate, besides being evidence that the midwife has satisfied the examiners, is at present a guarantee that she has passed through a satisfactory course of training. This would no longer necessarily be the case. Hence the certificate could not be unhesitatingly accepted as it now is either by public institutions or by individual members of the community—a result which would be obviously unfair to the midwives themselves, especially to those who had fully complied with all the educational requirements of the Central Midwives Board. The association therefore earnestly hopes the Council will realise how important it is that the education and practical training of midwives and the regulation of their practice should be under the entire control of the one responsible body, the Central Midwives Board. Only in this way can the Board administer the act uniformly and effectively, and adequately fulfil the purpose for which it was created—namely, to raise the standard of midwifery attendance upon the poor throughout the whole of England and Wales.

Dr. Francis Fremantle, medical officer of the Hertfordshire County Council said: As a county medical officer of health, whose duty it is to administer the Midwives Act over a wide area of town and country, I beg independently and most heartily to endorse the objections held by Dr. Cullingworth and the Association he represents to the amendments by the Privy Council of the rules now under consideration. At present the relations of County Councils and other local supervising authorities to the midwives working in Poor-law institutions are not clearly defined. But pupil-midwives in these institutions, especially in the larger towns, are hoping to earn their certificates from the Central Midwives Board, and to settle down in private practice. They will then come under the direct control of the local authorities and their officers, who are therefore concerned in the efficiency of their training. On this efficiency of training depend the degree of reliance hereafter to be placed on the midwife in her important vocation and the inverse amount of supervision necessary to safeguard the welfare of the public from the results of careless, ignorant, or mischievous midwifery. Under the Revised Draft Rules, before they were amended by the Privy Council, we could accept without hesitation the certificate of the Central Midwives Board; and, if maintained at the same standard, it would give us, soon after the year 1910, a definite minimum standard for the whole of midwifery practice under our control. The amendments proposed by the Privy Council introduce certain exceptions to the minimum standard required, certain gaps in the fence, to be filled by the Local Government Board at their discretion. These gaps constitute the greater part of the fence—namely, the whole thoroughness of the practical training, as guaranteed by the Poor-law superintendents of that training. There is left only such guarantee as is afforded by the examination. It is now commonly recognised that practical work is the most important part of medical training, and that the guarantee of a properly conceived curriculum is of higher value than the fallible and limited results of an examination. The certificate of the Central Midwives Board depends chiefly for its value on the curriculum it guarantees, and this guarantee, in the case of pupils trained in Poor-law institutions, it is proposed to transfer to the Poor-law authorities. In other words, it is proposed, in the case of these candidates, to make the Local Government Board responsible for their fitness, using only the organisation of the Central Midwives Board for the examination, and then to entitle the successful candidates to the title and privileges of the standard fixed by the Central Midwives Board. This must work out in one of two ways. Either the standard of the Local Government Board must be invariably equal to that of the Central Midwives Board, or it must occasionally differ. That the two standards should be invariably equal is inconceivable. To ensure the invariable observance of a practicable standard equal to that of the Central Midwives Board would require in the Local Government Board an advisory board of equal experience in matters concerned with midwifery. It cannot possibly be said that the present Poor-law medical staff of two inspectors for the whole of England and Wales, distinguished and hardworking as they are, can ever attain such experience. Granted then that the standards will be liable to differ, the difficulties of administration are at once doubled, and more than doubled. We who carry out the law will have to distinguish between the standard of the Central Midwives Board, which we can test and trust, and that of the Local Government Board, which we shall be unable either to test or to trust. The difficulties must be immeasurable; the hardship to certain midwives must be extreme. This last effect the next speaker will best explain. Finally, an exemption having been once granted to the authorities of the Poor-law, exemption would inevitably, and with far greater justification, be claimed, for instance, by the Army Nursing Service. Exemptions would become the rule, and the uniformity intended by the Act would be destroyed. We hope, therefore, that the revised rules, the object of which in the very wording of the Act is "to secure the better training of midwives and to regulate their practice," may be allowed to stand as drafted by the Central Midwives Board, and so left applicable to all midwives, whether working under the Poor-law or not.

Miss Brodie-Hall, Poor-law Guardian, Eastbourne, said: Though I am asked by the Association to touch especially on one or two points as a guardian, in which work I have had 25 years' experience, there is one point of quite general interest, which seems to me even more important, and that is the extremely undesirable moral influence on the community of any claim to exemption from the regulations of the Act being allowed. If certain conditions affecting the health and well-being of the community are considered so serious as to justify an Act of Parliament being passed to regulate them, surely it will throw discredit on those regulations if a prominent Government Department is allowed to claim exemption from the provisions of the Act—a Department not having even the plausible excuse of being a medical department, having indeed only two medical officers on its staff for the supervision of the 650 union infirmaries throughout England and Wales. We all know that the working classes who are chiefly affected by the Act are only too ready to evade any legislation on health or sanitation, and to disbelieve in its utility; and surely when they learn that a Government Department has exempted itself from the Act they will seem to some extent justified in their views. It will be disastrous if the efforts made to raise the standard of midwifery among the poor should be hindered by sanctioning any exemptions to the rules. The result would undoubtedly be the setting up of two standards of efficiency; for the Act assigns to the Central Midwives Board the duty of regulating what the training of a midwife should be, but the Local Government Board claims to train the midwife merely to its "own satisfaction," and that brings me to my second point—namely, the ease with which, I am sorry to say, and I speak from personal experience, guardians can evade the orders of the Local Government Board. That Department has hitherto made no rules or regulations whatever for the training of midwives, but if it had I would point out not only the possibility, but the great ease with which the orders of the Local Government Board are habitually evaded by guardians when they do not wish to comply with them, and where any restrictive policy needed to secure the best administration is constantly resented by them as "fads" or "red tape." Thirdly, there will result from this exemption great hardships to the midwives themselves. It is well known that Poor-law nursing is not so attractive as hospital and private nursing, and that nurses soon leave the service of the Poor-law and set up for themselves or to work in other institutions. It will be extremely hard on the midwife starting in business for herself that though she has, as she believed, been trained in accordance with the Act, she is really of a lower grade than others holding the Central Midwives Board certificate. She will not only be looked down upon by her fellow midwives on the higher grade, but, as a previous speaker has mentioned, she will be regarded with some doubt, if not suspicion, by the county and borough medical officers of health, under whose inspection she will then have to work, and they will require her to prove her trustworthiness before they will allow her to practise. Some, indeed, owing to insufficient training, may, I fear, be found unable to comply with the Central Midwives Board's requirements till they have undergone a further course of training—a great hardship and expense they could ill afford. In short, the midwives trained in Poor-law institutions will be heavily handicapped, and when this is once discovered by intending

### THE CENTRAL MIDWIVES BOARD AND THE LOCAL GOVERNMENT BOARD—*Continued.*

midwives they will give the Poor-law a wide berth, and so make matters doubly difficult for guardians, who already experience great difficulty in securing ordinary sick nurses for their infirmaries. With two authorities, each training to its own satisfaction, it will be found impossible to standardise the system of midwifery training.

Miss Alexander said : I have the honour to represent the Society of Poor-law Workers, a Society which was founded in 1899, and the objects of which are to bring together metropolitan guardians and others who have practical experience of the Poor-law, and to afford the means of giving organised joint expression to the views of experienced administrators of the Poor-law. The Society is anxious that the work done in workhouse and infirmary maternity wards should reach the highest possible standard, and we feel strongly that medical treatment under the Poor-law needs bringing into line with that provided by the best hospitals and voluntary charities. At present each Poor-law institution throughout the kingdom to a great extent creates its own standard, there is practically no outside criticism, no contact with public opinion, and very little touch in some cases with the advance of medical science. We are far from deprecating the work of the Local Government Board. Two medical inspectors, one for London, the other for the whole of England and Wales, can hardly be expected to come in contact personally with our large institutions, except at long intervals and in exceptional cases. We also place a high value on the work of the London assistant inspector, Miss Stansfeld, who is most anxious to improve and raise the standard of Poor-law work, and whose visits are extremely helpful in this direction. From the nature of the case, it is not to be expected that the competition for infirmary posts would be as keen as it is for the best hospital appointments. Guardians do sometimes—whether from carelessness or ignorance —appoint unsuitable officers to positions of great responsibility. When this is done the whole institution suffers, and the standard of work is lowered, to an extent which would not be possible in a voluntary hospital, where consulting physicians pay constant visits, and where consequently far less depends on the right selection of one medical officer. In the opinion of our Society, nothing but good would result from inspection by experts of workhouse and infirmary maternity wards, and both the standard of treatment of patients and the training of midwives would be uniformly raised. Well-organised institutions would certainly have nothing to fear from inspection, and would indeed welcome suggestions and criticism. The visits of the Commissioners in Lunacy to Poor-law lunatic and imbecile wards are a great and undoubted benefit. The placing of Poor-law schools under the inspection of the Education Department has lately raised the status of Poor-law teachers. The Society believes that it is most important that the training given to all midwives should be certified as thorough, efficient, and uniform, and feels strongly that it is essential that one standard should be maintained, and that no step should be taken which may lower the prestige of midwifery appointments under the Poor-law or detract from the status of those who hold them. As one who has during 12 years' experience as a Guardian of the poor for Kensington obtained an intimate knowledge of this subject, and having personal acquaintance with both hospital and district nursing, I fully endorse the views of the Society.

### A Discouraging Reply.

Lord Crewe, in reply, said : I welcome the visit of your deputation, and thank you for the clear way you have set forth your views, and also for the unexampled brevity with which you have been able to state fully the case you wish to lay before me. I want it to be understood that I entirely recognise the excellence of the work done by the Central Midwives Board, and that in anything I have to say by way of comment on what has been set forth to-day must not be taken as derogating in the least from a recognition of the public services it renders. But I confess I have heard with surprise it stated as a matter of fact that there is a project to set up another standard of training or examination for midwives. My perusal of the correspondence that has passed does not lead me to suppose that that is intended, and it is certainly not a project I should be disposed to countenance. But I must emphasise the fact that the Local Government Board is responsible—directly responsible to Parliament—for the conduct of its institutions, and therefore when it asks the acceptance of its assurance that the training of its midwives is the same, it has a right to expect that guarantee should be accepted. One of the speakers referred to the Central Midwives Board as a responsible body, but that is the one characteristic which it does not possess. The Local Government Board is responsible to Parliament, and the assurance as to the training of midwives in Poor-law institutions is one which the Central Midwives Board ought to accept. To speak of this training as inferior begs the whole question. It seems to be assumed that the training in Local Government Board institutions must be inferior, but I venture to think that when that is said the case is being somewhat overstated. As to one point made by the last speaker, I must point out that the Board of Education is a public department which the Midwives Board is not, and I therefore cannot admit any analogy between them. I trust that the difficulty which appears to exist is really an unsubstantial one, and more one of form than of fact. It is suggested that the Central Midwives Board—an irresponsible body—should take over the inspection of Poor-law institutions. I may say at once that I could not countenance such an idea, and I am quite sure the Privy Council would not give its consent to such an arrangement. The position is undoubtedly one of considerable difficulty, and one that was certainly not foreseen at the time of the passing of the Act which constituted the Central Midwives Board. That Act, for which those associated with me were not responsible, left out of account the possibility of a difference of opinion between the Board and the Government departments. I hope I shall not have to recommend any alteration in the duties or powers of the Board, bearing in mind, as I said in my opening remarks, the excellent work done, and the self-denying and unremunerated labour for which all must be grateful. I can assure you that I will give the matter the fullest consideration, but as to the suggestion for a system of inspectors of institutions of a Government character, which would involve the appointment of a large number of trained and expert officers, I cannot hold out any hope of agreement to that.

Sir Dyce Duckworth urged that it was the opinion of the large body of people, practical experts represented there, intimately acquainted with the Central Midwives Board, and also with the Local Government Board, that they had no confidence at all in the ability of the Local Government Board to train and examine midwives. They did not believe the machinery existed for the work, and the Local Government Board was totally unfit for it. Certainly the inspection desired would require a large number of suitable inspectors.

Miss Brodie-Hall asked permission to give one example from her own union—one of the best in the country. They had only one *bona fide* midwife, she had no certificate, but she was a capable nurse. The medical officer, finding she was competent, left all confinement cases to her. To that the Guardians put a stop ; but the Local Government Board allowed it—did not know of it ; indeed, had no machinery for knowing. If their suggestion for inspectors was not adopted, then the Local Government Board ought to provide itself with inspectors.

Lord Crewe said he quite understood that the second line of criticism was as to the methods adopted by the Local Government Board. He would only add that, assuming there were shortcomings, it would be quite possible to meet them in the way suggested by the last speaker—namely, by the setting up or improvement of the machinery.

The deputation then withdrew.

### The Royal National Pension Fund for Nurses.

### THE TWENTIETH ANNUAL MEETING.

The twentieth annual general meeting of the members of the Royal National Pension Fund for Nurses was held at River Plate House, Finsbury Circus, London, on Thursday, March 21, 1907, under the presidency of Sir Henry Burdett, K.C.B.

The Secretary (Mr. Louis H. M. Dick) having read the notice calling the meeting, and letters and telegrams of regret having been reported,

Sir Henry Burdett, in moving "That the report, accounts, and balance-sheet be received and adopted," said :—

### The late Mr. Edward Rawlings.

It is my painful duty this afternoon to record, on behalf of the Council and myself, with feelings of the deepest regret, the loss we have sustained by the death of Mr. Edward Rawlings. Mr. Edward Rawlings was associated with our Chairman, Mr. Hambro, in business for very many years; he had been a member of the Council of the Pension Fund from the commencement, and there has been no member in all that time who has given more attention to the work or shown more devotion to the interests of the nurses. We miss him because of his business qualities, of his regularity and punctuality in the discharge of his duties, and because of his personal charm. It will be well for this Fund if we can always find gentlemen like Mr. Edward Rawlings who will so fully and disinterestedly, and indeed whole-heartedly, give their time with infinite pleasure to enable the women workers in the nurses' world to make proper provision for themselves when they are past work and have to retire from duty.

### Sir Edgar Speyer, Bart.

I have the pleasure—and it is a great pleasure—to inform you that the Council will be strengthened by the gentleman who has consented to be elected in the place of Mr. Edward Rawlings—Sir Edgar Speyer, who is very well known in the City of London and equally well known in the United States of America as a man of great business talent, generosity, and wealth. By joining this Fund he will, if I may say so, practically complete the unanimous support of the great City houses who are now represented on the Council, so that we may claim that, financially, the Council is the strongest Board in connection with any commercial enterprise probably in the Empire. (Cheers.)

### Some Interesting Figures.

I must now ask your patience for a few minutes while I refer to the figures in the report. Taking the pension branch first, it is a great pleasure to be able to point out that there was an increase in the policies issued, which numbered 1,347 in 1906. Those policies will one day provide for the nurses £20,000 a year in annuities or pensions. The annuity premiums paid on the deferred policies amounted to £12,613; and, in addition to that, those nurses who have decided to buy immediate annuities—that is, an annuity which commences to be paid at once—have paid into this Fund in the last twelve months no less than £28,000. (Hear, hear.) Those figures may be taken to mean, of course, what the observer or the man of knowledge may think it well to deduce from them, but to me they mean this—that whatever else may be said for the working nurse, they do eloquently testify to this fact, that she stands prominently out in these days amongst women workers as the one class of woman who, with relatively very moderate emoluments, does her best, and does her best bravely, to provide for herself against the day of sickness and old age. (Hear, hear.) Our total premium income last year was £113,186. I consider that that is a wonderful result when you remember that eighteen years ago Miss Florence Nightingale, whom we all honour, and whom you nurses especially honour as your illustrious head, wrote to me about this Pension Fund in its early days, and asked me whether I really thought it was wise to go on with the movement, because the nurses had not enough money as it was to provide for their necessities, and she was afraid that it was quite impossible that they could save adequately to make the Pension Fund a real living thing. In eighteen years, however, we have a premium income of £113,000! That is the best practical answer that the nurses could have given.

### The Savings Bank Branch.

Now it is interesting to note another side of this Pension Fund which I have often dwelt upon, and it is the savings bank branch, as I always like to call it. It is a funny thing about this Fund that, as a matter of fact, though we would rather the nurses did not surrender their policies, still we realise that the withdrawals in fact represent a portion of the work of the Fund which testifies eloquently to its usefulness to the whole nursing body, for it is quite certain, as I am sure you will agree, that if it had not been for the Pension Fund most, if not all, of the money withdrawn would have been spent in the year it was earned, and there would have been no savings of the kind. Now, the withdrawals of last year by 371 nurses on 393 policies amounted to £24,757; that is an average sum of savings taken out by each of those nurses of £63. That in itself is an immensely useful side of the Fund, and it enables nurses to be much more independent than many ladies, because they very frequently withdraw on marriage, and owing to their savings in the Pension Fund they are able to provide themselves with their own trousseaux. That naturally appeals to a mere man like myself, who is also the father of a family and has daughters.

### Over 700 Nurses with Pensions.

The actual pensions distributed at the present time—that is, the number of nurses who are in receipt of annuities or pensions—is 718, of whom 100 are new pensioners during the year 1906. They received last year in pensions £16,000, and the average annuity for each nurse is £22. That is, unfortunately, a very small sum in one sense, but if you look at all the figures you will find that the average is very greatly reduced from the circumstance that there are a number of very old nurses who had to join late in life, and who are now becoming annuitants, and, of course, they had to enter for relatively small sums. This reduces the average very much, but I hope that the tendency is to increase the emoluments of the working nurses, outside institutions especially; and I am glad to see a gentleman present to-day who comes from the North, who takes a great interest in this branch of the work, and has brought about a movement in favour of helping the district nurse especially to procure pension and sick pay provision, which she certainly deserves as much or more than any member of the nursing body.

### More Nurses Require Sick Pay Policies.

In the sickness branch there were 144 new policies, and the amount distributed was £1,788, against a total premium income of £1,725. That is to say, the actual amount distributed in 1906 in sick pay was 4 per cent. more than the total sum received by the Fund from the whole of the sick pay policy-holders. We have been trying for years, with the aid of the Actuary, to get this sick fund on to a financial and actuarial basis which would leave us something in hand

### THE ROYAL NATIONAL PENSION FUND FOR NURSES—*Continued.*

after we had met all the claims for sick pay on behalf of the nurses. There is amongst the nursing body—and it is understandable—probably a greater amount of sickness than among the average women workers in other callings and in domestic life, but we have now brought this sick fund to a condition where we see our way to accomplish what we have been working for for eighteen years, and that is to put the sick fund in such a position that the premium income shall each year more than provide the whole of the demands upon it. (Hear, hear.) It would be quite ample now if the nurses as a whole would understand that there is no branch of the Fund's work which gives them a better return than a sick pay policy. I shall have something to say to you a little later on on this point, but I wish to emphasise most definitely the excellent financial investment which a sick pay policy proves to every nurse, whether she is working in an institution, on her own account, privately, or as a district nurse. We find in our experience that the successful and more intelligent nurses among our policyholders invariably take out sick pay policies, and that is a point which I should like you especially to bear in mind. We have, as you know, a reserve fund (I may as well explain this to you, or you may think we are getting into debt) of £34,934, so the difference which we have had to meet of some £60 this year does not trouble us at all. *Fourteen nurses received sick pay during every day throughout the whole of 1906.* That is an object-lesson for nurses, and one which they might take to heart, because who of us knows when our turn will come to be ill, or what will be the duration of the illness when the time comes? Then you may like to know that every participant for sick pay received on an average in 1906 nearly £7; 257 nurses altogether received sick pay, and 36 received sick pay on more than one occasion during the year.

### Over One Million Pounds Invested.

The invested funds amount to £1,103,441—an increase of £108,870 for the year. Those figures show that we must expect before many years go by to have £2,000,000, and, as time proceeds, many millions as the capital funds of this the greatest thrift agency among working women the world has ever seen. (Cheers.)

### The Economy of the Management.

Of course, those of you who joined first, and all of you who have joined this Fund at all, have shown by joining your confidence in the management. Now, I am speaking in the City of London, and confidence in the management from the shareholder's point of view must be based on strictly economical considerations. I am therefore very happy to be able to point out to you that the expenses of management of this Fund are only 4 per cent. on the premiums received, which were rather under £115,000. This rate of expenses, according to "Bourne's Assurance Manual," is below that of any mutual office in this country, although the premium income of the Fund, dealing with one group of women workers alone and being a relatively new society, is necessarily much less than that of any of the larger and older mutual offices. Then, again, when the expenses of the Fund are compared with those of the non-mutual offices they are shown to be in every case 100 per cent. less than those of the lowest non-mutual office, where the premium income is many times as great as that of the Pension Fund. (Hear, hear.)

### The Benevolent Fund.

We have another branch of this Fund which does a very great and useful work, and that is the Junius S. Morgan Benevolent Fund, founded, as you may remember, in memory of the man who, by coming forward foreseeing what this Fund could do, and backing that opinion by the offer of £20,000, if necessary, to enable it to be established, has done more for you probably than anyone else connected with the Fund. Now, this Benevolent Fund is excellently managed by a committee of matrons and ladies, of whom Lady Rothschild is the President; and it is a gratifying fact that they emulate the efforts of the Council by their attention to the work, for Lady Rothschild has never missed, I believe, a single meeting of this committee during the whole period of twenty years. (Hear, hear.) That, I think, is a striking testimony to the way in which the work is done. You have in Mrs. Farmer, the Secretary, a lady who devotes her time to visiting individual cases, and the work of this committee is especially good and valuable from the fact that it does not deal with any cases unless they know actually, from personal inspection, what are the real needs and necessities of each nurse, and then those needs and necessities are met in the most practical and adequate way. Now, the grants last year of this Fund were £965, and they are confined, as you know, to policy-holders in the Fund who, for some reason which they could not avoid, may find themselves in temporary pecuniary distress. The income of the Fund from interest and dividends was only £935, so that really it was £30 short of the actual money which was required to provide for the pressing and urgent needs of those policy-holders of the Fund who had met with misfortune. I wish to bring that point out quite definitely, because our Secretary, Mr. Dick, who, with his staff, devotes such energy to the work, is very anxious that each policy-holder should recognise that this Benevolent Fund is a means by which they may render in the days of their health a little personal service in the cause of their less fortunate sisters. Every year Mr. Dick sends out a note, in which he asks each policy-holder to give 1*s.* to the Benevolent Fund. This at first was very successful, for 750 nurses out of probably some 5,000 policy-holders subscribed 1*s.* Now that we have some 10,000 policy-holders, I regret to say that these 1*s.* subscriptions for some reason have fallen off, and that last year we only received 1s. from 566 nurses, or £28 6s., against an expenditure of £965. As I have mentioned, members of the public and others have given subscriptions to the Benevolent Fund amounting to £138, so the actual needs of all the nurses have been met without trenching on the capital. But I think it is quite reasonable (and I believe it would be done if we could make every individual nurse understand the position) to say that at least 80 per cent. of the nurses among our 10,000 policy-holders could perfectly well give 1s. a year to the Benevolent Fund, which would yield a revenue of £400. I commend that fact to your consideration, and I ask those present to talk it over with their sisters and fellow-nurses when they leave this room and in the course of the year.

### The Nurses' Pension House.

I now come to two interesting developments which have taken place in connection with the Fund during 1906. First of all, as you are aware, it has been decided to erect a Nurses' Pension House fronting the Embankment Gardens near Charing Cross. It will occupy the site of the house which was once occupied by Peter the Great, in which we found some wonderful ceilings and some very valuable and interesting oak panelling, which has been carefully preserved, and which, when the new building is erected, you will have the pleasure of seeing—I hope at the annual meeting next year. I mention that date because building operations in London are always difficult, and we have already experienced some delay, but if all goes well this building ought to be completed and roofed in by the end of the present year. If that is so, I shall hope that we may be able to have it ready for holding our annual meeting there in 1908.

Queen Alexandra Approves New Brooch and Armlet.

You are aware that the Queen has considered the question of the armlet, because I made a representation to her Majesty that these armlets are very inconvenient, and that nurses found in practice that they could not wear them habitually, so that they had practically to be kept for ceremonial occasions, and did not quite fulfil the purpose for which they were designed, which was to give every Pension Fund nurse something to show that she was one of Queen Alexandra's nurses. I wrote to the Queen and asked her whether she would object to our substituting a brooch for the armlet, and I received a reply in which Miss Knollys says: "The Queen regrets that the armlet does not answer, as she thought it a very effective and distinctive badge, but, such being the case, her Majesty is quite ready to accept the brooch as a substitute, and agrees with you that the largest one is much the best." Then I wrote and pointed out that as her Majesty seemed to have an affection for the armlet (and it is well you should know that she has, because she takes a great personal interest in you all as well as in this Fund), it was desirable that we should retain the armlet, and I therefore made a suggestion, which the Queen accepted in the following letter: "The Queen thinks that your last idea meets all difficulties, and willingly agrees to the brooch and armlet both being used according to circumstances—namely, the armlet for public and ceremonial occasions, and the brooch for every-day use." Mr. Dick has here the armlet and the brooch, which will be open to inspection after the meeting, although I expect that most of you know all about it.

Yorkshire's Co-operation and that of Insurance Officials.

I want, in conclusion, to say a few words about the great addition of membership from Yorkshire, especially from the West Riding, and Scotland. In the West Riding we have had a very large number of nurses who have joined the Fund during the year in consequence of meetings which have been held at which the Secretary has been present, and at which he has been assisted very materially by many gentlemen of influence locally. We have also had other co-operation, which it is a great pleasure to us to acknowledge, because it shows that this National Pension Fund is recognised in the insurance world, as it is recognised in the city of London, as standing apart from any mere ordinary business, that it has been conceived in a spirit and is conducted upon lines which render it impossible for any business undertaking to compete with it successfully as a matter of business. We therefore now have the co-operation of very many officials and insurance companies throughout the country, who help us directly by sending to us policy-holders and indirectly by advising nurses who apply to them to come to the Fund when it will be to their interest to do so. We are grateful for that co-operation, and we think that those members of the insurance world who are doing this work are assisting us in a very practical and excellent way to help the nurses to help themselves.

The Prudential Assurance Company and Mr. T. C. Dewey.

But the greatest and the most material assistance we have had from the insurance world has come from that great company, the Prudential Assurance Company. It is a marvel of organisation, and I take it that it is no exaggeration to say that its intimate relations with a multitude of people in these islands is one of the most remarkable developments in insurance that the world has ever seen. I was advised to-day by a gentleman, who has gone very fully into this matter and who has inspected the unique organisation of the Prudential Assurance Company, that there is no undertaking in the world that can really compare with this company either in America or elsewhere. We are very much indebted in many ways to the Prudential, because it has branches all over the country, and wherever it has a branch, there the Pension Fund has a friend. (Hear, hear.) But the Prudential Assurance Company has done more than this. It has given us Mr. T. C. Dewey, Fellow of the Institute of Actuaries, as a member of our Council. I am sure my colleagues will endorse my words when I say that his joining the Council has been momentous for good in the interest of the conduct of this business in every direction. We are very much indebted to him, and we feel that the work he has done for you as a member of the Council is probably more valuable than that rendered by any other member. (Cheers.)

The Secretary and His Staff.

The Secretary during the year has visited many parts of the country, and held many meetings, and as the result—as I told you—we have had a larger number of policies taken out in 1906 than ever before in the history of the fund. That shows the zeal and ability of Mr. Dick and the fact that he is the right man in the right place. (Hear, hear.) We as a Council gratefully recognise this fact, and we desire to-day, in thanking Mr. Dick, to thank also all the members of his staff, who, individually and collectively, do their work exceedingly well, as I am sure Mr. Dewey, who keeps a very close eye upon the working of the office, will be able to testify much more authoritatively than I can.

Wanted a few Real Men and Real Women.

Now, it has struck me in connection with these visits that every Hospital Committee and every Institution Committee has a duty in this matter. We will do everything we can to give information and to make it easy for nurses to save their money by joining the Pension Fund, but what we want is a man or a woman—a real man or a real woman—in connection with every committee, every institution throughout these islands, who will take the trouble to understand the Pension Fund and all it means to the working nurses. When they have done that we know they will cheerfully render a little personal service, by never resting till all the staff working in the institution of which she or he is a member join the fund and so put their savings into it. I throw out that as a suggestion, and hope that it may be fruitful in results.

Sick Pay, Nurses and the Workmen's Compensation Act.

To revert to the sick pay, I want to say a word about the Workmen's Compensation Act and the nurses. It has been thought by many nurses, as they are included under this Act, that there is no necessity for them to insure for sick pay—that if anything happens to them in the course of their work, they can come down on the hospital or the institution or the home, and that the latter will have to pay. Well, of course, that would be a very happy state of things, in one way, for nurses, but I am afraid, if you relied upon it, you would be relying upon a broken reed. The Act, I have reason to believe, at first did not contemplate the inclusion of nurses at all. They were excluded on the ground (or it was proposed to exclude them on the ground, I understand) that they were connected with philanthropic institutions, which could not possibly provide under the Compensation Act for their nurses. Well, this was a plausible idea so far as it went, but although the nurse is often a part, and a most important part, of a philanthropic institution, she is not therefore a philanthropist. As a working nurse

### THE ROYAL NATIONAL PENSION FUND FOR NURSES—*Continued.*

she is a working woman, and has to earn her living like every man amongst us, and to think that she was to be excluded because she was devoting the best years of her life to attendance on the sick, and that that attendance is mostly carried out by what are known as philanthropic institutions, was once seen to be absurd so soon as the facts were represented by the right authorities, so that nurses were struck out of the schedule and came under the Act. What is their position under the Act? Their position is clearly defined in these words : "Under the new Workmen's Compensation Act all nurses, probationers, and servants in the employment of the hospital fall within the scope of the Act in respect to accidents." Only as to accidents, mind you; keep that in your mind. "It was a first thought that the hospital might also be liable in respect of diseases contracted by attendants from patients during the discharge of their duties." Now legal advice has been taken on this point by some of the hospitals, and I have also consulted the solicitors of the fund, and they have given the opinion "that with respect to diseases, the Act was directed against diseases known as industrial—*e.g.*, phosphorus poisoning, and that the hospital would not be liable therefore for nurses." It therefore comes to this—that, as far as I can make out, the only disease for which hospitals may be liable on behalf of their nurses engaged in nursing in a public institution is anthrax, because they do occasionally get cases of anthrax. All the diseases are mentioned in the Act, and anthrax is a highly contagious disease, and the nurses may possibly contract it; but, considering the fact that a case of anthrax is never seen in many hospitals and but seldom seen in a few hospitals, I do not think any nurse who has any pretension to be provident or to regard herself with the respect with which a nurse should regard herself would be contented to trust to her chance when ill of getting emolument from her employer under the Workmen's Compensation Act. Therefore, I come back to my point, which is that those nurses who have not joined for sick pay should look into the matter and make up their minds to take out a sick-pay policy, because the premium is so relatively small as to prove a matter of very little importance to most of them.

### Their Majesties the King and Queen.

I have had great pleasure in submitting the reports of this Fund to the King and Queen, and on March 4 Lord Knollys wrote: "His Majesty was very much interested in the report of the Pension Fund, and directed me to express to you his great satisfaction that the Fund is progressing so favourably. His Majesty thinks that the scheme has certainly been a great success." The Hon. Charlotte Knollys wrote under date March 3: "Her Majesty was most gratified with the report, and acknowledges to the fullest the wonderful work that has been done on behalf of the nurses through the Pension Fund, which is one of the greatest successes of the day, both as regards charity and finance." I believe you will agree that when we look back to the commencement of this Fund, and when we note the extraordinary success which has attended its establishment, we may look forward to the future with every confidence and hope that, as the years go by, the Fund will grow stronger and more important. It should thus become an ever-increasing strength and consolation to every nurse who has to obtain her living as a working woman in these islands. (Cheers.)

### "Greater Value for Money than in any other Company."

Mr. Thomas C. Dewey, F.I.A., General Manager Prudential Assurance Company: Ladies and gentlemen,—The figures in this report are eminently satisfactory, and I think it is a matter of congratulation that the results of the year should have been so successful. (Hear, hear.) I feel that there may be some policy-holders who do not yet realise the exceptional advantages that are conferred by the Pension Fund, but it is a matter of congratulation that so many of the nurses really invest their savings—which, as the Chairman has said, are practically provision for old age—in an institution of unquestioned stability—(hear, hear)—and in an institution which gives greater value for the money received than any other company in the kingdom granting annuities. It always seems to me a matter of regret that there should be so many nurses still outside the Fund. (Hear, hear.) I believe that we have not yet reached half the nurses in England.

The Chairman: About one-third.

### "Speak about the Fund."

Mr. Dewey: The Chairman says about one-third. That is because they really do not realise what the Fund is, and, if I may say so, I think that the nurses themselves who are policy-holders are apparently nervous—perhaps bashful—in speaking to others. Now what a power for good these 700 annuitants should be—they who are receiving quarterly payments, and payments that have been increased from time to time by bonus additions which they really never anticipated. Yet if it depended on our nurses' recommendations, I am afraid that the number of new policies this year would have been very small. To our excellent Secretary, Mr. Dick, must really be given the credit for this extraordinary increase in business. I do not know whether you realise that during the year the new premiums received amount to more than £40,000—not half the assurance companies in London can show a new premium income of £40,000—and these large figures are due principally to our Secretary—(hear, hear)—for he has not only controlled the office most efficiently, but to my knowledge he has travelled during the past year all over the kingdom, and has attended upwards of 40 meetings, to explain the advantages of joining the Pension Fund. I do hope that the nurses and their friends will for the future not leave everything to the Secretary, and I urge them very strongly to take some part in extending a knowledge of the advantages to be obtained by taking out policies in this institution. I have very great pleasure, Mr. Chairman, in seconding the adoption of the report. (Hear, hear.)

The Chairman: Ladies and gentlemen,—The resolution is "That the Report, Accounts, and Balance-sheet be received and adopted."

Those of opinion that that should pass will please signify the same in the usual way. On the contrary. Carried unanimously. It is customary at these meetings to delay any remarks which you may have to make until we have got through the formal business. I hope that we shall have many speeches a little later on, but—I do not know why—it has always been the custom to get through the formal business first. I have now to present to you the result of the scrutineers' report on the voting for the policy-holders' representatives on the Benevolent Fund Committee. The following is the report of the scrutineers:—

"We hereby certify that we have examined the ballot cards received for the voting for the annuitants' and policy-holders' representatives. 1,544 cards were received, 242 were spoiled or contained more than two names.

The following is the result of the voting: Miss S. A. Swift, 1,025; Miss F. Smedley, 779; Miss Mary M. Macfarlane, 618; twenty-two ladies received amongst them 36 votes.

We are informed that 7,490 cards were sent out."

Mr. Nairne will propose the next resolution.

A Very Material Fact.

Mr. Perceval A. Nairne: The resolution which is entrusted to me is one for the re-election of the retiring members of Council. The statement which has been made by the Chairman of the continued progress of the Fund and of the very fine position it has been brought to is a testimony to the work which the Council have done, and the work is sufficient in itself to induce you to re-elect the members of the Council who have served you so efficiently in years past. I should like, on behalf of the members of the Fund, to add a word to what has fallen from the Chairman in expression of the sorrow of the members at the death of Mr. Edward Rawlings, to whom we were so much attached, and with so much reason. Everyone who has met him knows the interest he took in the Fund, and the great attention he paid to it at all times down to the day of his death. It has been a matter of gratification to me, and I am sure to the members of the Fund also, to hear from the authoritative voice of Mr. Dewey that the Fund presents greater advantages than those afforded by any other institution in the kingdom which grants annuities. This is a thing we have been trying to impress on all interested in the nursing profession during the whole period the Fund has existed. It has been denied frequently by what I suppose we may call some competing institutions, but we are now able to put forward on such high authority as that of Mr. Dewey that the advantages offered by the Fund are really greater than those to be obtained by nurses elsewhere. (Hear, hear.) I move: "That the members of the Council who retire by rotation—namely, Mr. Alfred C. de Rothschild, Sir John Watney, Mr. Thomas Bryant, F.R.C.S., Dr. Herbert P. Hawkins, and Dr. S. H. Habershon—be re-elected members of the Council."

Dr. Potter on Nurses' Salaries and Sick Pay.

Dr. George W. Potter: In seconding this motion, I should like to say one or two words to the assembled nurses. I am a little disappointed that more of them do not join the sick-pay branch. No one, I suppose, knows a tenth part of the working of the Sick Fund (not even Mr. Dick) as well as I do—that is, its innermost working; and no one sees half as much as I do of the distress that nurses suffer who have no provision when they are sick. I have been specially struck by one fact—that is, the number of nurses who suffer from very serious heart trouble—not necessarily valvular disease—long before they reach the age of 50—trouble which disables them for five or six weeks, three months, six months, a whole year, or longer. The proportion of nurses suffering in this way is to my mind very large indeed, and quite unexpected. I have one remedy to propose. It must not be thought that it is the nurses' fault always that they do not join in very large numbers; I think it is to a large extent the fault of the people who pay them. My suggestion is that nurses should have at least 25 per cent. larger pay than they get on an average; and I wish very much—I do not say this on the spur of the moment to please the nurses, but after reflection—that the Pension Fund could initiate some movement for such a general increase of pay as I have suggested. I know the character of nurses, their general standard of education, their devotion to duty, and the disastrous effects upon some of them resulting from that devotion; and I say that if they were paid 25 per cent. more than they are, they still would not be paid anything like what they ought to be. I suggest that if 25 per cent. more pay were given to them, every nurse could join the Pension Fund and join the almost more important sick-pay branch. I leave it to your consideration. I have great pleasure in seconding the resolution.

Re-election of the Auditors.

The Chairman put the motion, and, having declared it carried unanimously, said: I have now to move the re-election of the auditors, Messrs. Whinney, Smith, and Whinney. These gentlemen have performed their work to the entire satisfaction of the Council for very many years, and it gives me great pleasure, therefore, on behalf of the Council, to propose their re-election.

Mr. R. Ernest Alexander: I have the pleasure of seconding that.

The Chairman put the motion, and, having declared it carried, said: I have now to inquire, in accordance with the agenda, whether anyone in the audience would like to make any remarks? I hope there will be many.

"A Nice Little Sum."

Miss Newton, lady superintendent Nurses' Home, Ipswich, said: References have been made several times this afternoon to the smallness of the expenses in connection with the Pension Fund as compared with other offices, I think I can illustrate that by telling you of what occurred to me not long ago. I was told one morning that a gentleman wished to see me urgently. I found a little man with a black bag and a very fluent tongue; he spoke for ten minutes before I could get in a word edgeways; he was eloquent on the disadvantages of the Royal National Pension Fund, and the superiority of his own particular office over it. I explained that I was a member of the Fund, and I knew that we had at least one advantage over other offices, that our working expenses were smaller than other companies, and there was consequently a greater profit for the nurse. "Oh! quite so, madam we run ours on the same style; now, may I ask you not to discourage your nurses from joining my society, and should you induce any to join you would not be asked to do it for nothing, I would give you a nice little sum for every fresh member you got me."

"Now how very good of you," I said. "Do you know I have induced many nurses to join the Pension Fund, but Mr. Dick has never paid me a penny. Will the commission you pay me come from out of the working expenses, or be paid by some benevolently disposed person?"

He smiled faintly, took up his bag and left.

Hospital Committeemen and the Benevolent Fund.

Miss Chambers (Ancoats Hospital, Manchester) suggested that the committees of the various hospitals in the kingdom should have their attention drawn to subscribing to the Pension Fund and the Benevolent Fund. The Chairman of the hospital which she represented at Manchester had already become a subscriber, and she understood that three other hospitals in Manchester were ready to follow this example if other institutions could be induced to do so. She thought it would probably encourage the nurses if they saw that their own hospitals were helping the Pension Fund and the Benevolent Fund.

Pay the Nurses Better.

Mr. Pollitt: I just want to ask a question, but, before doing so, I should like to make one or two observations on what has passed. In the first place I am sure that to be a member of your Council must be one of the most pleasing things in life. You have attained great results, but those results are not what they ought to be in consequence of the poorness of the nurses' pay. I think that it ought to be just three times what it is, and I have conceived a very great affection for the doctor who has spoken about an increase of 25 per cent. I share that view, certainly, but what I say is that the advance should be on the £100, which a nurse costs, not on the £30 cash that is paid to herself. As to the gentleman who called upon this lady who has addressed us,

THE ROYAL NATIONAL PENSION FUND FOR NURSES—*Continued.*

I advise her to give him a good shaking if he calls again, and I think she would be able to do it. (Laughter.) Mr. Dick's work I have watched in Lancashire, and I quite endorse all that has been said about him. I am quite sure that you would do well in this Fund if you encourage him, by recognising the very great services rendered by Mr. Dick in carrying out the directions of the Council, not only in the letter but in the spirit. In Lancashire there is a very general feeling that the time in nursing work has arrived for taking a lunar, as Captain Cuttle said, of the whole work. All consideration appears to be for the patient and extremely little for the nurse, on whom everything depends. Of course, the patient must be considered, but are we to go on always considering the patient at the expense of the nurse? I do not think it admits of discussion at all that up to now nurses have not been adequately remunerated.

The Chairman: With respect to what you have said, I should like to point out that this question of pensions is a question of wages or salary—earning power—and the question cannot be divorced from the question of earning power. That is the point we have to bear in mind. I agree with you that the emoluments—the pay—to a large number of nurses in this country are far too small. I consider, however, that what Dr. Potter asks is as much as we can hope for, and perhaps rather more than we shall get for some years to come. As to the reference which has been made to "a nice little sum," I am very glad that the Matron from Ipswich was good enough to give that experience, because it is a fact—and nurses ought to know it—that there are men and women going about who are mean enough to try and persuade nurses to put their savings into particular offices in order that they themselves may make a commission every year out of the premiums such nurses may pay into it. In this Fund every penny you pay is yours, and every penny it will earn you get. You get it because we are a mutual office and our working expenses, as I have said, are so small. We have had experience in the past of persons connected with nursing papers who had made arrangements to receive "nice little sums" as Miss Newton has explained. I think, therefore, that every nurse will be wise to bear these facts in mind, and to take the advice of someone who has a knowledge of business before she chooses some other kind of investment than that which is offered to her by taking out policies in this Fund. As to Mr. Pollitt's suggestion that the Benevolent Fund should be increased three times, supported as it was by the Matron of Ancoats Hospital, who suggested that the public should have the opportunity of subscribing, if the Fund were in that position, we could go outside our own Fund, but at present we are confined to it. We should be very much obliged to you if you would advocate the general public giving contributions to the Benevolent Fund.

Mr. Pollitt: I will do so, but London is the seat of Government, and we look up to London, and we should like to see London lead the way.

The Chairman: But at the present time Lancashire is the seat of prosperity. (Cheers and laughter.)

Well Done. Much Good and More to Follow.

Mr. Bryant, F.R.C.S.: I rise to move that we should unanimously pass a vote of thanks—and a very warm vote of thanks—to the Chairman for the way in which he has conducted the business of the meeting. I listened to his statements with very great pleasure, as I always do, for I want to remind you that our Chairman was the founder of this institution. (Cheers.) Indeed, while I was listening to the lucid statement he brought before you, and pointed out the good work done by the Fund, and urged you to press still more upon others the advisability in their own interests, to follow in your footsteps and join the Fund, I thought how proud he must be at the position which the Fund has attained; for I can well remember the doubt which those who were associated with him had in its origin as to the possibility of the Fund's becoming a great success. He has told you, however, to-day that £1,100,000 odd are now invested—all for you—and you cannot forget that the working capital of the Fund was only £20,000. I myself feel proud at having been connected with an association which has from its origin been so prosperous, which has already done so much good, and which is calculated to do so much more good in the future. I therefore put it to you that you will kindly give a hearty vote of thanks to the Chairman for all the great work he has done on your behalf.

The vote was accorded with the utmost enthusiasm by the meeting.

The Chairman: Ladies and gentlemen, I thank you very much. I am sure that you have heard quite enough from me this afternoon already, and the Secretary has written this important note on his blotting-pad—"that tea is getting cold." (Laughter.)

The proceedings of the meeting then terminated.

### Meeting of the Central Midwives Board.

A meeting of the Central Midwives Board was held at Caxton House on Thursday last, Dr. Champneys in the chair. There were also present Miss Wilson, Miss Paget, Mrs. Latter, Mr. Parker Young, and Dr. Dakin. The minutes of the meeting of February 28 and the special meetings of March 7 and 8 having been passed, the Board proceeded to deal with the correspondence.

There was a letter from the Secretary of the Midwives' Institute, stating that Dr. Stanley B. Atkinson, barrister-at-law, had been elected to represent the Institute on the Board for the year ensuing March 31, in place of Dr. Dakin. On the motion of the Chairman, seconded by Mr. Parker Young and heartily supported, the Board expressed to Dr. Dakin their regret that he finds it impossible to continue his attendance, and their unanimous thanks for his valuable services in the past.

A letter was read from the Clerk of the Council signifying the Board's power to remove names from the Roll without allegations of misconduct.

Another letter from Dr. Niven, of Manchester, reported a conviction, under Section 1 (1) of the Midwives Act, of a woman who had been exposing a plate announcing herself as a midwife without any qualifications for so doing.

A recommendation was carried that a letter from Dr. Sidney Barwise, County Medical Officer for Derbyshire, as to the licensing and inspection of certified midwives taking cases into their own homes, should be referred to the Act Amendment Committee when formed.

After consideration of a special report by Dr. Boxall and Dr. V. Bonney on the February examination, it was decided that the attention of the examiners be drawn to Rules E. 16 and E. 19 (*a*) (note), and that no further action be taken in the matter.

On the presentation by the Secretary of the financial statement for 1906 and the report of the auditors, it was announced that the fee of 20 guineas for a monthly audit offered by the Board was objected to, and it was agreed that the matter should stand over.

An application from Sussanah Davies, No. 1706, for the removal of her name from the Roll on the ground of defective hearing and memory was granted.

Application for Approval as Training School.

The Committee recommended that the Leeds Maternity Hospital be not approved; but an amendment by Miss Paget was carried, and was to the effect that the Secretary of the Leeds Maternity Hospital should be recommended to apply again at a future date, when the work of the training school was more consolidated.

Applications for Approval as Teacher.

The application of George Martin Fox, M.B., D.P.H., was approved, and that of Samuel Craddock, M.R.C.S., L.S.A., who is the Poor-law Medical Officer at the Bath Infirmary, was postponed until the question of Poor-law institutions should be settled.

Applications for Approval to Sign Forms III. and IV.

Angela Rosamund Griffiths and Rosa Maria Charlotte Ross were approved. Mary Lydia Houlding's application was refused, and several applications were postponed for further inquiries.

Mr. Parker Young then asked the Chairman, on behalf of the public, which the Board represents, to make a statement as to the position the Board now stands in with reference to training in Poor-law institutions. This statement was embodied in a written record of the whole controversy between the Board and the Privy Council.

A motion by the Chairman, seconded by Miss Wilson and carried, was that Mr. Parker Young be added to the Finance and Office Management Committee.

The next meeting of the Board was fixed for April 18. The Standing Committee will meet on April 11, and the hearing of penal cases has been arranged for May 16.

### Guy's Hospital Nurses' League.

ANNUAL MEETING.

On Friday evening the annual meeting of the Guy's Hospital Past and Present Nurses' League was held in the Nurses' Home. There were present many nurses who had trained at Guy's and now hold responsible positions as matrons or sisters in other hospitals. The chair was taken by Mrs. Fisher, an old Guy's sister. Naturally there were many enthusiastic greetings as one friend met another and chatted over times past, and to allow of this tea and coffee and light refreshment were served at 7.30 p.m. One could not fail to see the beneficial effect of this yearly meeting as experiences and knowledge on various subjects were vigorously exchanged.

At eight o'clock the meeting began with reading the minutes of the previous year, and then followed the result of the ballot for sisters and nurses—one of each—representing each branch of the Recreation Society. Votes of thanks to retiring members were passed, and the new members were welcomed. A subject keenly discussed was that of a suitable badge for nurses belonging to the League. Suggestions were invited, and from some of the most practical and suitable badges will eventually be prepared and submitted for criticism by members.

There were two or three short speeches. The Chairman said that it was due to the energy and wisdom of Miss Swift, the matron, that the League had first started and was still in a flourishing condition.

Miss Swift observed that it gave her genuine pleasure to see so many of her old nurses again, and she hoped that each year they would be able to come and meet each other. Although she had seen her way clear to establish the League, still, when the time came for her to be succeeded by another, she had no doubt that still more plans would be thought of for the benefit of the profession. After a few more remarks the matron sat down amidst hearty applause. Miss Smith, the Secretary, also received a very cordial vote of thanks for her indefatigable work during the past years for the League.

Combined with the meeting the third annual exhibition of photographs took place. One of the sitting-rooms in the Nurses' Home made an excellent showroom, and Mr. Hollier, of photographic fame, kindly came during the afternoon and acted as judge. There were many entries, and these were divided into Classes A, B, C, D, and E, the latter, however, not being eligible for prizes, because it consisted of work not done exclusively by the exhibitor. Miss Smith and Nurse A. Lloyd were among the prize-winners. The successful subjects by Miss Smith were :—In Class A : Landscapes, 1st prize, "Birch and Bracken"; 2nd prize in Class B : Portraits, "Tea in Chelsea Hospital"; 2nd prize in Class D : Postcards, "O'Connell's Monument, Dublin"; and a special prize in Class C : Architecture, "The Henriette Raphael Home, Guy's." Nurse Lloyd took 2nd prize for a set of postcards in dog studies.

After the exhibition had received its share of attention, and the meeting being over, a number of good slides were shown from the Royal Photographic Society, demonstrating the state of perfection which may be attained in photography. The very pleasant evening finished about 10.30, and all agreed that it had been the best meeting yet held.

### Queen Alexandra's Imperial Military Nursing Service.

We are officially informed that Miss M. Willes, Miss A. P. Wilson, Miss E. Fraser, Miss M. H. Graham, and Miss F. Macpherson have been appointed staff nurses in Queen Alexandra's Imperial Military Nursing Service. Miss M. Mark, sister, has been transferred to the Royal Herbert Hospital, Woolwich, on her arrival from Malta; and Miss N. Blew, to the Cambridge Hospital, Aldershot, from the Military Hospital, Portsmouth. Miss V. C. Paschali, staff nurse, has been transferred to the Queen Alexandra Military Hospital, Millbank, London, from the Military Hospital, Chatham; Miss A. P. Wilson has been appointed to the Royal Infirmary, Dublin; and Miss C. E. A. Harries, to the Connaught Hospital, Aldershot. Miss E. M. Rentzsch and Miss H. Hartigan have been transferred to Malta from the Royal Herbert Hospital, Woolwich; Miss M. Brown and Miss S. Richards, from the Queen Alexandra Military Hospital, Millbank, London; Miss M. B. Williams and Miss M. J. Hepple, from the Cambridge Hospital, Aldershot. Miss M. Mark and Miss W. E. Massey, sisters, have arrived from Malta and Egypt respectively.

### Presentations.

Darlington Hospital.—Miss Hunt, lady superintendent of the Darlington Hospital, on giving up her work at that institution, has been presented by numerous friends with a cheque and a handsome travelling clock; by old patients with a gold chain and pendant, by the nursing staff and others with a travelling clock; and by the domestic staff with a gold and pearl safety-pin brooch.

### Everybody's Opinion.

[Correspondence on all subjects is invited, but we cannot in any way be responsible for the opinions expressed by our correspondents. No communication can be entertained if the name and address of the correspondent are not given as a guarantee of good faith, but not necessarily for publication. All correspondents should write on one side of the paper only.]

### A PROBATIONER LOSES HER SIGHT.

"A Sympathiser at Bridgewater" writes: I enclose postal order for 2s. 6d. for the unfortunate nurse who recently lost her eyesight at West Ham Infirmary.

"A Paris Nurse" writes: I enclose five shillings for the nurse who lost her eyesight by an accident in nursing an enteric fever patient.

### A LONG-FELT WANT

"Schwester" writes from Germany: Having suffered more or less from corns on my feet during the whole of my nursing career, I feel deep sympathy with "Anxious." I found great relief from painting the corns every night and morning with a mixture of salicylic acid, collodion, and Indian hemp (Martindale); by doing so regularly I was then able to cut away the horny layer of skin easily when I had soaked the feet in hot water every few nights. I pay strict attention to the footgear—as you wisely suggest—and always have a good kid lining put in both boots and shoes; when night nursing I find it better to wear a thin pair of kid shoes with heels, and a larger pair of felt shoes over these.

### POOR-LAW INFIRMARIES—THE SELECTION OF MATRONS.

"Fairplay" writes: In the filling up of vacancies for matrons in Poor-law infirmaries in London, I have been surprised at the number of hospital-trained nurses who have been appointed. This seems a little unfair to the infirmary-trained nurse, more especially as it is a well-known fact that hospital authorities will not appoint or even consider an application for any vacancy from a nurse with infirmary training. If Boards of Guardians are to give their best appointments to hospital nurses what is the use of training? In future there will be great difficulty experienced in obtaining suitable probationers for Poor-law, as there appears little or no chance of rising in the profession; and even in the infirmaries where the matron is hospital trained the nurses have the same difficulty in obtaining the higher posts.

### THE DISTRICT NURSE AND THE DOCTOR.

"E. W." writes: "Justice" seems to imagine that I may be to blame. In what way I fail to see. Was it my fault that I fell ill with influenza, and when ill had I not a right to proper medical advice as well as other people? "Justice" appears to think that nurses ought to pay for medical advice; there was no question of money about it. If the doctor had sent in his bill he would have been paid. I consider he treated me very badly in not coming when sent for, and then getting offended because another doctor was called in. I have nearly all the ladies on the committee on my side, and they have been most kind, and think I was right in what I did. "Justice" is, perhaps, one of those fortunate people who has met with doctors as they should be, both to nurses and to everyone else. If she would come and work under this doctor in less than twelve months she would most likely alter her opinion. My reason for making the matter public was not so much on account of myself, but for the benefit of other nurses seeking a similar post in order to show how awkward it is to be so dependent on one doctor for cases.

### Appointments.

[No charge is made for announcements under this head, and we are always glad to receive and publish appointments. The information, to insure accuracy, should be sent from the nurses themselves, and we cannot undertake to correct official announcements which may happen to be inaccurate. It is essential that in all cases the school of training should be given.]

General Hospital, Weston-super-Mare.—Miss Alys E. Parsons has been appointed sister. She was trained at the General Infirmary, Chester, where she has since been sister. She has also been sister of a Leeds surgical home.

High Wood School, Brentwood.—Miss Lillian E. Fincke has been appointed charge nurse. She was trained at Royal Albert Hospital, Devonport, and has since been sister at North Devon Infirmary, Barnstaple; sister at the General Hospital, Ramsgate; and charge nurse at Gore Farm Hospital, Dartford.

Hospital for Diseases of the Throat, Golden Square, London.—Miss Jessie Smith has been appointed matron. She was trained at King's College Hospital, London, and has since been sister at the Royal Chest Hospital, City Road; out-patient sister at the Children's Hospital, Shadwell; and assistant matron at the General Hospital, Nottingham.

Nurses' Co-operative Home, 86 Lower Leeson Street, Dublin.—Miss Cherry has been appointed lady superintendent. She was trained in Sir Patrick Dun's Hospital, Dublin. Miss Cherry has also been appointed secretary to the Irish Nurses' Association and to King Edward's Benevolent Fund for Nurses in Ireland.

Redhill Cottage Hospital.—Miss Bridget M. Powell has been appointed staff nurse. She was trained at the Royal South Hants Hospital, Southampton, and has taken sister's holiday duty.

Sick Asylum, Colindale Avenue, London.—Miss Caroline Amelia Lee has been appointed sister. She was trained at Woolwich Infirmary, and has since been charge nurse and pupil midwife at West Ham Union Infirmary. She has also done private nursing.

West Bromwich District Hospital.—Miss Flora G. Pegg has been appointed matron. She was trained at Guy's Hospital, London, and has since been charge nurse at the New Hospital for Women, Euston Road, London; staff nurse at Netley House, Henrietta Street, Cavendish Square, London; theatre sister and deputy matron at Wolverhampton General Hospital; and matron of Salop Infirmary.

### TRAVEL NOTES AND QUERIES.

By our Travel Correspondent.

Holland for Easter (A. T.).—I hope you will see this in time, but you have left the question too late: First return from Harwich, *via* The Hook, £2 15s. 11d.; second return, £1 18s. 7d. to Amsterdam; first return, £2 5s.; second return, £1 9s. to Rotterdam, but your best plan is to write to Messrs. Cook, Ludgate Circus, and ask for a circular ticket, *via* Rotterdam, to include Gouda, Amsterdam, Hoorn, Alkmaar, Haarlem, Leyden, The Hague, and Delft. Arrange the week thus: One night at Amsterdam (not stopping at Rotterdam) to see the pictures, churches, etc., one at Hoorn, one at Alkmaar, one at Haarlem—see Leyden *en passant* by taking an early train from Haarlem, and three nights at The Hague. This leaves you one night to spare, which might be profitably spent at Amsterdam, visiting the Island of Marken. You will see a long list of hotels for this tour given to a correspondent last week, so I will not repeat it. I know of no apartments at The Hague, and pensions are no use unless you remain some time. Go there to Hôtel du Passage. The money is in florins—one florin equals 1s. 8½d. Ask for rooms on third floor always.

### Notes and Queries.

**REGULATIONS.**

**The Editor is always willing to answer in this column, withou any fee, all reasonable questions, as soon as possible.**

**But the following rules must be carefully observed.**

1. **Every communication must be accompanied by the name and address of the writer.**
2. **The question must always bear upon nursing, directly or indirectly.**

**If an answer is required by letter a fee of half-a-crown must be enclosed with the note containing the inquiry.**

*Cairo.*

(247) Can you tell me the name of a nursing home in Cairo? I have nursed in the Riviera.—*Joan.*

We do not recommend private nursing homes. Possibly, however, the matron of the Kasr-el-Ainy Hospital, Cairo, might forward your application to a suitable quarter.

*Uniform.*

(248) May a nursemaid attendant wear nurses' uniform though untrained?—*The Garlands.*

Unfortunately nurses' uniform is often usurped by nursemaids, as it is not illegal to do so. It is, however, extremely bad taste to wear the garb of a profession to which you do not belong.

*Home for Crippled Man.*

(249) Do you know of a home for a man who has lost one leg and has neurosis of the arm?—*Sympathetic.*

There are numerous charities supported by trade societies which might suit the case. Consult "Burdett's Hospitals and Charities," price 7s. 6d., from The Scientific Press, 28 and 29 Southampton Street, Strand, London, W.C., or from any bookseller.

*Epilepsy.*

(250) Is there a home where an epileptic young man can be received?—*Heaton.*

Write to the Home for Epileptics, Maghull, near Liverpool; the County Epileptic Colony, Ewell, Surrey; and the David Lewis Epileptic Colony, Watford, near Alderley Edge, Cheshire; asking, if unable to receive the case, where they advise your applying.

*Home for Defective Woman.*

(251) Will you help me to find a home for a poor woman of 26, who is slightly defective in intellect? A little could be paid for her.—*Birmingham.*

Write to Miss Wemyss, Washwell, Painswick. She may be able to help you.

*To Train as Nurse.*

(252) I am in domestic service and wish to train as a nurse Where can I go?—*Fareham.*

Get "How to Become a Nurse," price 2s. 4d. post free, from The Scientific Press, 28 and 29 Southampton Street, Strand, London, W.C., or any bookseller; and write, as there directed, to any hospital you prefer.

*Nursing in India.*

(253) Can you tell me where I can get information as to private nursing in India. I have had four years' general training, two years' fever nursing, and one year's experience in private nursing?—*Chester.*

Your training is all that can be desired. We do not give addresses of private homes, so that I fear your only course is to apply to the Secretary, Indian Nursing Association, Simla; though at present all vacancies have been filled.

*Home for Cancer Case.*

(254) I shall be glad if you can help me to find a home for a consumptive woman entirely without means.—*Bristol.*

Write to the sister in charge, St. Michael's Home, Cheddar. This home is under the St. Peter's Sisterhood, and is free. St. Catherine's Home, Grove Road, Ventnor, Isle of Wight, and St. Barnabas's Home, Torquay, require payment of 10s. a week.

**Handbooks for Nurses.**

| | Post Free. |
|---|---|
| "How to Become a Nurse: How and Where to Train." | 2s. 4d. |
| "Nursing: its Theory and Practice." (Lewis.) ... | 3s. 6d. |
| "Complete Handbook of Midwifery." (Watson.) ... | 6s. 4d. |
| "Preparation for Operation in Private Houses." ... | 0s. 6d. |
| "The Nurses' Enquire Within." ... ... ... ... | 2s. 3d. |
| "Nurses' Pronouncing Dictionary of Medical Terms" | 2s. 0d. |

Of all booksellers or of The Scientific Press, Limited, 28 & 29 Southampton Street, Strand, London, W.C.

### For Reading to the Sick.

EASTER.

ALLELUIA! Alleluia! Hearts to heaven and voices raise;
Sing to God a hymn of gladness, sing to God a hymn of praise.
He who on the Cross a Victim for the world's salvation bled,
Jesus Christ, the King of Glory, now is risen from the dead.

Christ is risen, we are risen; shed upon us heavenly grace,
Rain and dew and gleams of glory from the brightness of Thy Face,
That we, with our hearts in Heaven, here on earth may fruitful be,
And by Angel-hands be gather'd, and be ever, Lord, with Thee.

*Bishop Wordsworth.*

Because I live, ye shall live also.—St. John xiv. 19.

Easter Day comes as a bright day of sunshine after a stormy week. There are many devout Christians, thank God, to whom this last week has been a great deal more than an annual commemoration of historical events in a remote past. And Easter stands out, therefore, to them as the day when the King holds His Own again; a day of triumph after suffering, of glory after shameful indignity. . . . Jesus Christ comes into this world again straight from the frontiers of the other; the long, outward procession is stopped, and one traveller comes back as if there were no frontier, and no barrier. It is their Master Who had been away, their Saviour Who had died, their friend Whom they had longed to retain with them, Who stands in the midst of His Apostles; no new Incarnation, no fresh presenting of Himself to the world; it was the same Jesus, Who could pass backwards and forwards, and could carry a human body through the grave and gate of death. . . . Easter will shed its radiance over the tombs and grassy graves, where the spring flowers, the first fruit of earth's yearly resurrection, mark the last place of meeting between those who are left and those who have gone before; the furrow in which the seed is sown in dishonour to be raised in glory, in the hope which needs no demonstration, in the assurance which needs no proof, that when we meet again, we shall meet as friends those whom we parted with as friends; in the bond of a friendship which lasts for ever, where all friendships will be knitted up in the union of the great Friend, in loving Whom we shall also love one another.

My brethren, if friendship is eternal; if the grave is powerless to rend asunder those who have been knit together here in the fullness of mutual love, is there not a call to us, in friendship as in everything else, if indeed we are risen with Christ, "to seek those things which are above?"—*Canon Newbolt.*

Present with Thee!—Beseech Him for thy dearest,
And while thou bringest Him their every need,
Be ready when He sends thee to His nearest
To tell them that "The Lord is risen indeed."

Present with Thee!—for those in Jesus sleeping
Plead with a glad and grateful heart to-day,
Yield them to Him Who holds them in His keeping
To bring them forth when we shall rise and they.

*Anon.*

